# Caries of the Acromion Process

**Published:** 1844-04-06

**Authors:** 


CARIES OF THE ACROMION PROCESS.

Mr. Fergusson, of King's College, has recently removed the entire acromion process of the left scapula for caries. When he commenced his operation, he supposed that he should meet with a small portion of bone, nearly or altogether loose, but he soon discovered that the whole process was affected, and required removal. The gouge and curved cutting pliers were the instruments used. The patient did well.

